# Home and Pay Hospitals

**Published:** 1890-04-26

**Authors:** 


### Home and Pay Hospitals.

### MR. BURDETT ON THE PAY SYSTEM.

At the twelfth annual meeting of the Home Hospitals Association, held last week at Fitzroy House, Fitzroy Square, at which Mr. Henry C. Burdett, the vice-president, took the chair, an interesting resumé of the progress of the pay system in England was given by the chairman.

### The Home Hospital.

Mr. Burdett pointed out that the Home Hospital was established for the accommodation of persons who were prepared to pay at a remunerative rate for their treatment in hospitals, that the institution was to depend for its support entirely upon the monies derived from patients' payments, and that for twelve years this system had proved an entire success financially, although they had reduced their rates more than once since the Home Hospital was first founded. Every class of person mentioned in the original prospectus as being eligible for admission had had representatives under treatment during the year, with the exception of governesses, and no less than 77 eminent medical practitioners had been in attendance at Fitzroy House. Although nine other institutions of a similar character had been established within a stone's throw of the Home Hospital, the effects of competition had not been felt, and as a matter of fact more patients had been treated during 1889 at Fitzroy House than in any previous year. He would be glad if any lady or gentleman interested in the pay system, and having time at their disposal, would communicate with him should they desire to be identified with the management. He would specially welcome the assistance of a gentleman of intelligence and ability as an additional hon. secretary, who would thus have an opportunity of learning hospital administration thoroughly, and of acquiring information and experience which must prove valuable.

He then went on to consider how far the Home Hospital had leavened the whole system of hospital administration in this country by its example and success. He mentioned incidentally that a dispute which was causing much attention in Paris at the moment was very apposite to that meeting. This dispute was due to the hostility which had arisen to the sisterhoods who formerly nursed patients in the Parisian hospitals, and so created an intense desire to prove that the foundation of hospitals was anti-Christian. It was remarkable and interesting to note that there were many points of resemblance between the Pre-Christian Hospital and the Home Hospital itself.

### Early Hospitals.

The first real hospital was one situated at Epidaurus, and dedicated to Æsculapius. This hospital was in full working order five hundred years before Christ. It consisted not only of a general hospital but of two special hospitals in addition. The Temple proper contained accommodation for ordinary or acute cases, and also a hydropathic establishment. In separate buildings were a lying-in hospital, and a chronic hospital, and to the latter the aged and infirm, and those believed to be *in articulo mortis*, were admitted. The priests of Saturn in Egypt treated people for lunacy fifteen hundred years before Christ. This last fact is important, because we find that under them lunacy was considered as a disease not to be repressed, but as one to be overcome by kindness and skilled treatment, and the temples of Saturn combined much that is now to be met with in our best lunatic asylums. These hospitals and asylums were founded on the pay principle, for no one who resorted to them had so little self-respect as to desire to obtain free medical relief, and all took care to compensate those at whose hands they received treatment and benefit. There were home hospitals at Rome 250 years before Christ, called *Tabernae Medicæ*, which at first only contained consulting rooms, where the physician used to see out-patients on payment. After experience it was found that there were many cases that could be better treated if they were constantly under observation. So rooms were built for the reception of in-patients as *annexes* to the *Tabernæ*, where paying patients were admitted and treated successfully. This is a very interesting point, because these *Tabernæ* included all the branches of our present hospitals, *i.e.*, an out-patient department, an in-patient department, consulting rooms, and hospital accommodation for paying patients, but all those who attended, *i.e.*, both the in-patients and out-patients had to pay for the treatment they received.

### The Spread of the Home Hospital System in England.

He would now turn to England, and see how the home hospital system had been spread abroad. Twelve years ago hardly any of the existing medical institutions would entertain the question of payment by patients. There were then very few of these institutions that had paying patients, and it was pointed out that with the exception of cottage hospitals, which received twelve per cent., only two or three hospitals received one per cent. of their total revenue from this source. In 1877 they were told at a meeting at the Mansion House and also in the newspaper press, that their greatest difficulty would be the financial one. They, however, determined to so start the Home Hospital, that it should pay its way from the outset, and that was what they had proved to be possible. It had invariably paid its way, and so it had come to be a sort of tower or pillar on one side of the river of life, there being on the other side a second pillar—the voluntary hospital. Between these two pillars, their object had been to build a bridge—the paying ward. It would be interesting to see whether their success had been a real success, and had left a permanent mark on the institutions of England. They might say it had, for after going through the reports of 159 institutions situated in London, the provinces, Scotland, and Ireland, he found that out of that number 78 hospitals now admitted paying patients and received a portion of their revenue from that source. They were distributed as follows:—out of eleven hospitals, with medical schools, in London, five had received payments from patients; in Scotland the whole of those with medical schools did so, and in Ireland three out of four. Of the eighty general hospitals in the United Kingdom thirty-seven admitted paying patients; and of fifty-two special hospitals, twenty-nine did the same. On looking at the figures more carefully he found that in London five per cent. of the income was derived from patients' payments and in the provinces fifteen per cent.; while in Ireland nine per cent. came from contributions from patients. Among the London hospitals which had adopted this system were St. Thomas's Hospital and Guy's Hospital, whilst the London Hospital had passed a special act enabling its committee to receive pay patients. But there was another more remarkable fact still—the Metropolitan Poor Law Infirmaries were leaning to the pay system. By a recent order of the Local Government Board, accidents and urgent medical cases were admitted by the medical superintendent if brought to the infirmary, and the guardians afterwards exacted payment of such a sum as might be mutually agreed upon. From the Home Hospital situated in Fitzroy Square for the sole accommodation of paying patients the cause had increased in public favour till the Metropolitan Poor Law Infirmaries, many of which were as complete as the large voluntary hospitals, had yielded to the humanitarian cry that no hospital should refuse admission to any case of emergency, and so the guardians now ask payment for those who can afford to make it.

Working Classes and Hospital Letters.

Thus payment by patients might one day become the *panacea* for hospital abuse. The artizan classes desired to see abolished all hospital letters whatever, and to have an opportunity of contributing to the hospitals. The most remarkable example they had of this wish was to be found at the North Staffordshire Infirmary where the artizan classes contributed so large a sum annually that they paid for the actual cost of all the patients they sent to the hospital, *i.e.*, both for themselves and the members of their families when ill, and at the end of the year they were able to give, in addition, a sum of upwards of £500 as a free gift to the institution to defray the cost of beds occupied by other residents in the Potteries who were not so fortunately circumstanced as themselves. Further, they had an example in the Hospital Saturday Fund in Birmingham, where this movement had been a greater success than anywhere else in the country, and where the system was to make a weekly collection at each workshop, and to place it in a box. When any employé of the firm was ill the foreman took out of the box a sufficient amount of money to pay for the cost of treatment at the particular hospital the patient wished to enter, and at the end of the year, on the eve of Hospital Saturday, the box was opened and all the money that was left was given as a free gift to the hospitals on behalf of the working men engaged in each workshop where this system prevailed. The Birmingham plan, to his mind, was a better one than that which established a central council, which collected the letters in exchange for the funds which were given by it to the hospitals, and then distributed these letters in any way they thought fit. The best, happiest, and only suitable arrangement was for the workshops to come into direct communication with the hospitals, and in that way the best results could alone be secured both to the working men patients who resorted to the hospitals, and to the hospitals themselves.

The Hospital Committee of the House of Lords.

Mr. Burdett said he had been led to make these remarks, having regard to the fact that a Committee of the House of Lords had been practically appointed, which would sit in a few days, and that the managers of this institution and others would be summoned before it to give an account of its work. They should, therefore, look ahead and see how they stood with reference to the many questions which they would thus have to face. Most people must agree, having regard to the fact that nearly 50 per cent. of the hospitals now derived an income from paying patients, that the influence of the Home Hospital had been distinctly satisfactory, and was a matter upon which they could legitimately congratulate themselves. Much of that success was due to the fact that in the early days they had had the support of Mr. John Walter, who was a firm friend, and believed in the cause of pay hospitals, and also that of the press generally, and especially of the *Standard* and the *British Medical Journal*. These newspapers, despite all the criticism they went through in early days, always remained firm friends, and to-day he thought it was only right to call upon them to join in these mutual congratulations on the undoubted success the Home Hospitals Association had secured.

